# Commentary: GABA Depolarizes Immature Neurons and Inhibits Network Activity in the Neonatal Neocortex *In vivo*

**DOI:** 10.3389/fphar.2015.00294

**Published:** 2015-12-10

**Authors:** Yuri Zilberter

**Affiliations:** Institut de Neurosciences des Systèmes, Inserm UMR_S 1106, Aix-Marseille UniversitéMarseille, France

**Keywords:** GABA-action, inhibitory GABA, excitatory GABA, neonatal GABA, depolarizarion, hyperpolarization, *in vivo*, *in vitro*

The study of Kirmse et al. (2015) provides long-awaited evidence that GABA acts as an inhibitory neurotransmitter in the neonatal brain—the same way it acts in the adult brain. To recap, the concept of GABA acting as an excitatory neurotransmitter in the neonates dominated research for a long time. Based almost exclusively on the *in vitro* studies (Zilberter et al., 2010; Bregestovski and Bernard, 2012), extrapolations have leads to the theory of excitatory-to-inhibitory switch of GABA action and its central role in brain maturation (Ben-Ari et al., 2007, 2012).

In anesthetized P3-4 mice, Kirmse and colleges first showed that GABA application induced a dramatic, 16-fold drop in cortical plate (CP) cell membrane resistance. Then, using non-invasive patch recordings (cell-attached), the authors found that applied GABA did not generate action potentials (APs) in any of the cells. To avoid confounding factors such as anesthetics and brain damage, the authors employed Ca^2+^ imaging on N_2_O-sedated animals keeping the dura-mater intact. In line with electrophysiological data, afferent electrical stimulation readily evoked AP-generated Ca^2+^ transients in the majority of cells while the cells were nonresponsive in the presence of glutamate receptor blockers, indicating that GABAergic transmission failed to induce APs. In line with these results, the epidural GABA applications failed to induce detectable Ca^2+^ transients in the vast majority of CP cells in P1 and P3-4 mice.

To clarify the mode of GABA action on network activity, the authors simultaneously recorded correlated field and fluorescence (recorded in some cases through the intact skull in the absence of sedation) responses to spontaneous activity in the intact brain. Recordings revealed spindle-like oscillations resembling spindle burst activity previously described in the visual cortex of neonatal rats (Hanganu et al., 2006; Colonnese et al., 2010). Interestingly, the oscillations were not affected by bumetanide (NKCC1 transporter antagonist which modifies the driving force for Cl^−^ currents via GABAA receptor channels) confirming the absence of GABAergic contribution to the spontaneous network excitation. However, diazepam (benzodiazepine strongly increasing the GABAA receptor channel open time) and gabazine (antagonist of GABAA receptors) strongly modified spontaneous activity inducing its inhibition and amplification, respectively. These results are in contrast to those in slices showing inhibition of spontaneous activity by GABAA antagonists [e.g., blockade of so called giant depolarizing potentials, GDPs, by bicuculine and picrotoxine in the original study (Ben-Ari et al., 1989)]. Altogether, the paper results show convincingly that GABAergic transmission provides efficient inhibition of network activity in neonatal mice—definitively the major message of the Kirmse and colleges study.

Regarding the mechanism of inhibition, the authors demonstrated powerful shunting induced by GABA in glutamatergic cells. Another potential factor in observed GABA action is a shift in membrane potential. Indeed, in the process of membrane depolarization during neuronal excitation, GABAergic transmission becomes progressively hyperpolarizing due to increasing driving force for Cl^−^ inward currents. In neonatal slices, however, the values for reversal potential of GABA-induced currents have been found to be so positive in respect to the resting potential (Ben-Ari et al., 2007), that GABA application in neurons could both induce generation of APs and efficiently promote spontaneous network activity (GDPs). The concept of excitation/inhibition switch in the GABA action during early development was created exactly on this background. Kirmse and colleges showed that *in vivo*, the GABA-induced shift in the membrane potential is either hyperpolarizing or depolarizing (in the majority of cells). The depolarization, however, was of a very small value since neither GABA application nor afferent stimulation evoked detectable Ca^2+^ transients in the vast majority of cells. Therefore, *in vivo* GABA-induced depolarization is radically different from that found in slices and does not contribute to the cell excitation.

Meanwhile, based on the authors' data, it is not possible to conclude that GABA action in P3-4 differs qualitatively from that in P1 or P25-27. Indeed, the larger number of GABA-induced Ca^2+^ transients at P1 vs. P3-4 (11/117 vs. 6/204) can be easily explained just by a higher membrane resistance of P1 cells, in contrast to the author's suggestion of higher intracellular Cl^−^ concentration at P1. As compared with mature cells, hyperpolarization was found in only one cell from 10 in P25-27 vs. 2/15 in P3-4, making any age difference unconvincing. In addition, comparison of the potential shift values does not have any particular meaning since this method (cell-attached patch) does not provide quantitative measurements (Mason et al., 2005). The evident common feature of all mouse groups was that the potential shifted by GABA was very close to the membrane potential at rest. Therefore, the data provided by the authors so far support neither the hypothesis of the depolarization/hyperpolarization nor the high [Cl^−^]_*i*_/low [Cl^−^]_*i*_ developmental switch.

Taking into account a small value of GABA-induced potential shift at rest and the absence of significant difference in GABA action between age groups, it is surprising that the authors focused on the issue of “depolarizing GABA.” A shift of few mV above or below membrane potential at rest provided by GABA has an unclear physiological implication and, as the authors agreed, is unlikely to make any significant contribution to the cell excitability.

Therefore, association of this shift with the very concept of depolarizing GABA is misleading since the accurate term for neonatal GABA action has always been “excitatory GABA” (Ben-Ari et al., 2007). The excitatory GABA concept has been seriously questioned in several studies (Rheims et al., 2009; Holmgren et al., 2010; Dzhala et al., 2012), which attempted to find out the reason for such “unusual” GABA behavior in slices. These studies suggested that the increased neuronal [Cl^−^]_i_ in slices may be the consequence of acute injury of neuronal processes or/and inappropriate metabolic conditions. They showed that correction of these abnormalities [e.g., intact hippocampal preparation used by Dzhala and colleges or enhanced energy metabolism in Rheims et al. (2009) and Holmgren et al. (2010)] resulted in GABA-induced inhibition of spontaneous network activity. Importantly, in respect to the resting potential, GABA was slightly depolarizing in the majority of neurons (e.g., Holmgren et al., 2010) and therefore the categorization of these studies as opposing “depolarizing” (Kirmse et al., 2015) instead of “inhibitory” GABA is factually incorrect.

To conclude, the study by Kirmse and colleges convincingly demonstrated GABA to be an inhibitory neurotransmitter in both developing and mature animals, which should have a big impact in the field of developmental neuroscience. Many important questions are still left for future studies, e.g., what is the developmental profile of [Cl^−^]_*i*_ and how it relates to the resting membrane potential. However, citing the co-authors' previous publication on the subject, “…an absence of GABA-mediated excitation (Rheims et al., 2009; Holmgren et al., 2010) could have major implications for a central hypothesis of developmental neurobiology” (Kirmse et al., 2010). Now, the authors give us the opportunity to reconsider this central hypothesis.

## Conflict of interest statement

The author declares that the research was conducted in the absence of any commercial or financial relationships that could be construed as a potential conflict of interest.
